# On the mean path length invariance property for random walks of animals in open environment

**DOI:** 10.1038/s41598-022-24361-9

**Published:** 2022-11-17

**Authors:** Federico Tommasi, Lorenzo Fini, Stefano Focardi, Fabrizio Martelli, Giacomo Santini, Stefano Cavalieri

**Affiliations:** 1 Dipartimento di Fisica e Astronomia, Università di Firenze, Via Giovanni Sansone 1, 50019 Sesto Fiorentino, Italy; 2 Istituto dei Sistemi Complessi, Consiglio Nazionale delle Ricerche, Via Madonna del Piano 10, 50019 Sesto Fiorentino, Italy; 3 Dipartimento di Biologia, Università di Firenze, Via Madonna del Piano 6, 50019 Sesto Fiorentino, Italy

**Keywords:** Biological physics, Statistics, Animal behaviour

## Abstract

Random walks are common in nature and are at the basis of many different phenomena that span from neutrons and light scattering to the behaviour of animals. Despite the evident differences among all these phenomena, theory predicts that they all share a common fascinating feature known as Invariance Property (IP). In a nutshell, IP means that the mean length of the total path of a random walker inside a closed domain is fixed by the geometry and size of the medium. Such a property has been demonstrated to hold not only in optics, but recently also in the field of biology, by studying the movement of bacteria. However, the range of validity of such a universal property, strictly linked to the fulfilment of equilibrium conditions and to the statistical distributions of the steps of the random walkers, is not trivial and needs to be studied in different contexts, such as in the case of biological entities occupied in random foraging in an open environment. Hence, in this paper the IP in a virtual medium inside an open environment has been studied by using actual movements of animals recorded in nature. In particular, we analysed the behaviour of a grazer mollusc, the chiton *Acanthopleura granulata*. The results depart from those predicted by the IP when the dimension of the medium increases. Such findings are framed in both the condition of nonequilibrium of the walkers, which is typical of animals in nature, and the characteristics of actual animal movements.

## Introduction

Random walk is at the basis of many phenomena in nature that span from neutrons scattering^1^ to light transport in turbid media^2–11^, random lasers^12,13^, photovoltaic devices^14–17^ and photon propagation in atmosphere^18,19^, to animal behaviour^20^, such as insects migration^21,22^. In addition, among the different kinds of random walk, phenomena such as anomalous diffusion and Lèvy walks have been also studied in light propagation^23–25^ and in harvesting strategies of animals^26–28^. In biology, the movements of animals have always attracted the attention of scholars through the centuries for their variability and unexpected features. Since movement is the first mechanism used by animals to respond to environmental challenges and to find resources, a full comprehension of its properties may help unveil many details of ecological dynamics^29,30^. Molluscs can prove an interesting subject to investigate the mechanisms and determinants of movement patterns, due to their slow motion, the limited spatial extent of displacement, and their reduced behavioural repertoire^31,32^. In particular, recent investigations have revealed interesting fractal properties of molluscan movement patterns. The displacement of several different species, from mussels to gastropods, have been analysed as being described by Weierstrassian Lévy walks, one of the simplest random walks that do not satisfy the central limit theorem^33^. Recent investigation on the movement of bacteria, for example, showed that in the absence of external flow and concentration gradients the movement paths are influenced by the level of confinement and geometrical complexity^34^.

Despite their completely different origin and nature, all these phenomena, under the conditions of homogeneous entrance of the walkers and without any preferred input direction, share an outstanding property: the mean path length $\langle L\rangle$ followed by a random walker in a delimited region of a broader environment is fixed by the geometry and size of the region, whatever the nature and complexity of the paths followed by the random walkers^35–38^. In a two dimensional medium the following very simple formula holds:

1 $$\begin{aligned} \langle L\rangle _{IP}=\pi \frac{S}{P} \end{aligned}$$

where *S* and *P* are the surface and the perimeter, respectively. It is worth to stress that such simple formula holds for any shape. This result has been also extended, in the field of photonics, to inhomogeneous media with non-uniform scattering and mismatch of refractive indexes^1,39–43^ and also generalized to demonstrate an equivalent invariance relation for the scattering of waves in resonant structures or even in Anderson localized systems^37,44^. This counterintuitive result, named also Invariance Property (IP) of Scattering, can be thought of as an extension of the mean chord theorem first introduced a century ago by Dirac^45^. Experimentally, such property has been confirmed in optics^39^ and, in connection with the presented work, in the biological context^46^, where bacteria randomly move within different disordered structures of different complexity. The hypothesis used for Eq. (1) is the constancy of the specific intensity of walkers, i.e., of the number of walkers that flow through unitary length perpendicular to a direction within the unit angle, at the boundary and inside the medium. The specific intensity and the walkers’ density are related, in particular, if one quantity is constant, so is the other, see, for instance, in the field of light transport refs.^4,40,41^. This condition is assured by the requirement of statistical equilibrium, whose meaning is that the quantities, characterizing the transport phenomenon, are constant. For animal walkers, the condition is verified in an enclosed environment where the animals are expected, after a transient time, to be randomized and characterized by a constant density.

**Figure 1:**
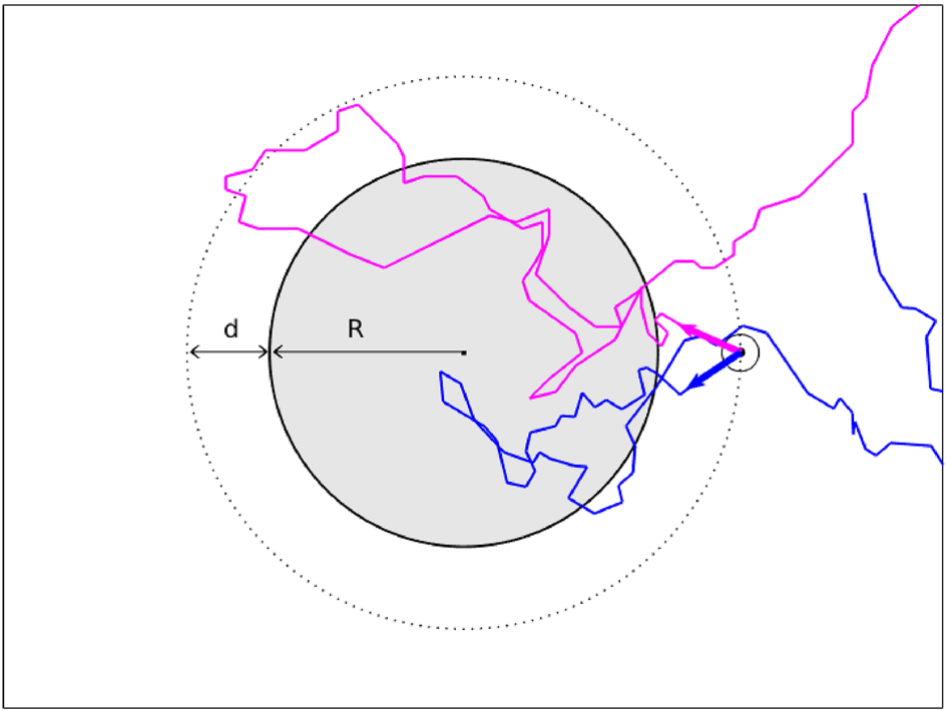
Schematic representation of the virtual medium. $R_e=d+R$ is the radius of the boundary source of animals. Two real trajectories (in pink and blue colours) are reported as example.

**Figure 2:**
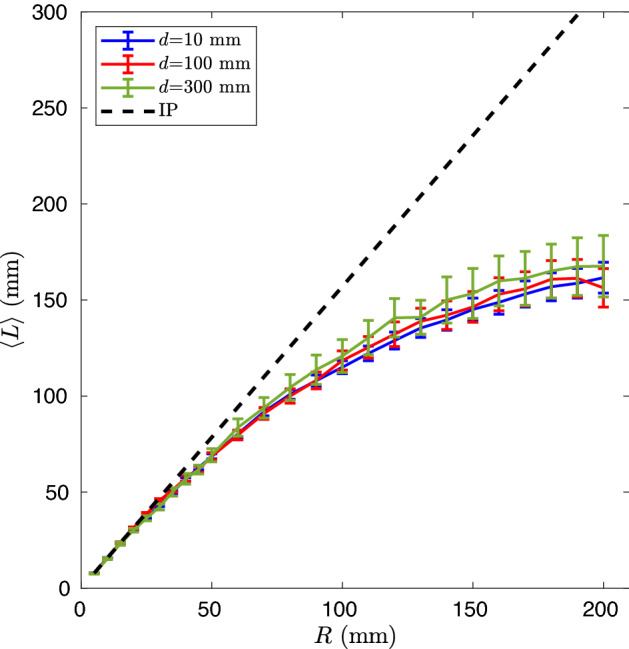
Average path length of the animals inside the inner circle of radius *R*. In the legend the values of the distance d from the external source circle. The mean free path $\ell _{MFP}$ is about 50 mm.

Great theoretical effort has been done on various aspects and implications of the IP. On the contrary, the experimental studies are few and very recent. In the field of photonics, the first experimental evidence has been reported in diffusive media^39^, whereas the generalization of IP has been recently investigated in contexts where the wave nature of light plays a fundamental role^44^. Another experimental result, in a completely different context and near to the content of this paper, has been reported by Frangipane et al. where the relation  (1) is tested for the movements of *Escherichia coli* bacteria^46^. Here we present a study that, using actual movements of animals registered in nature, analyses the mean path length $\langle L\rangle$, within a virtual medium where animals are introduced in an open environment without artificial enclosure. This open system-approach is not a priori in a condition of statistical equilibrium of the walker inside the medium, and indeed the out-of-equilibrium condition is the norm in the case of the animal behaviour in nature. The aim of this paper is to test the condition of validity of the invariance property in an open environment and its dependence on the extension of the analysed region and on the distribution of steps of the walkers.

**Figure 3:**
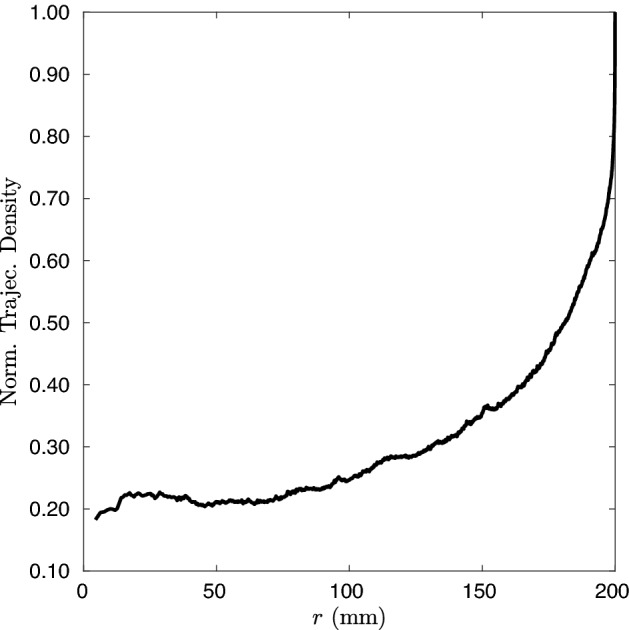
Trajectories density, inside the external source circle for $R_e$=200 mm, versus the radius *r* normalized at $r=R_e$.

**Figure 4:**
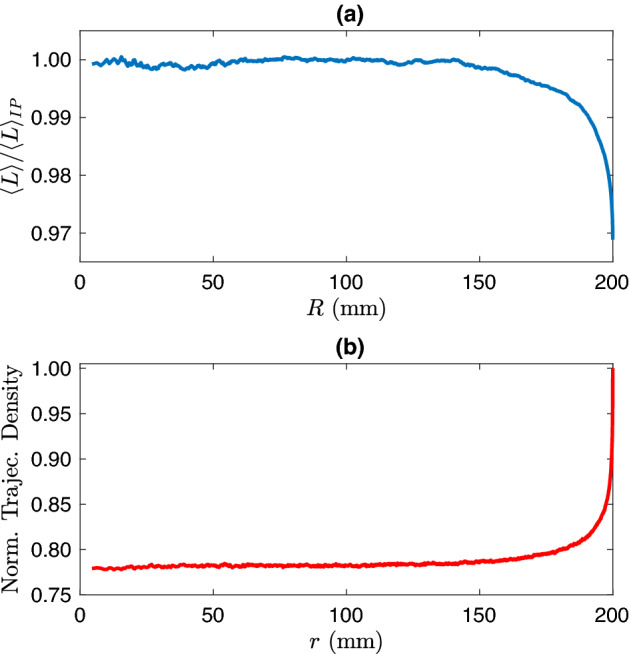
The simulations have been carried out by using an exponential path step distribution, a mean free path of 50 mm and $R_e$=200 mm. (**a**) Average path length of the synthetic walkers inside the inner circle of radius *R* normalized to the IP value. (**b**) Trajectories density for synthetic walkers: the values are reported versus the radius *r* and normalized at $r=R_e$.

## Results

We used the foraging trajectories of the chiton *Acanthopleura granulata* (Mollusca, Polyplacophora), a rocky-shore algal grazer that feeds on epilithic and endolithic microalgae^47^. The foraging behaviour of this species is described as ranging pattern, which means that its foraging excursions are randomly oriented and the animals do not home to a fixed starting point. The animal paths were recorded in an open field, following the methodology described in the Method section. The virtual medium considered for the analysis is schematically reported in Fig. 1. The open system consists in a circle that is the source of animals at distance *d* from an inner circle of radius *R* which is the domain under study. Any point of the external circle, of radius $R_e=R+d$, is a source of animals, with the path, starting in the point, obtained by a rigid translation of any registered path. In Fig. 1, for the sake of clarity, is reported a single point source that will be replied throughout the entire circle. The total length *L* is measured for all paths in the inner circle of radius *R*. We then performed the calculation of $\langle L\rangle$ for several values of radius *R* and distance *d* (details of the calculation are given in the “Methods” section). The results, reported in Fig. 2, show that $\langle L\rangle$ grows following the IP law ($\langle L\rangle _{IP}=\pi \frac{R}{2}$) for small *R* values while it deviates from it for large *R*. The dependence on *d* is weak. We would like to stress here that the calculation of $\langle L\rangle$ does not involve any modelization of the distribution of path steps of the animal movements because we use directly the registered paths. This procedure is unaffected by the unavoidable arbitrariness of performing a fit to extract the path step distribution $p(\ell )$ from data. In this text the symbol *L* indicates the length of a total path of the random walker inside the medium, whereas $\ell$ is the one of a single “step” along the path. An independent analysis of the paths with the methodology described in^48^ has been done to extract the characteristic mean free path $\ell _{MFP}$ of the animals trajectories. The resulting value of $\ell _{MFP} \simeq 50$ mm can be assumed as a scale factor for the size *R* of the region under study. For $R\ll \ell _{MFP}$, the results show that $\langle L\rangle$ is very close to $\langle L\rangle _{IP}$ (as expected for a quasi-ballistic case and the mean chord theorem also described by Dirac in nuclear physics^45^) while, for $R>\ell _{MFP}$, $\langle L\rangle$ is clearly smaller than $\langle L\rangle _{IP}$. One of the hypotheses for the validity of the invariance law is that the entrance of the walkers into the region under study must be of the Lambertian type, i.e., the amplitude of the entrance angle probability of walkers across the boundary must have a cosine dependence. Actually we introduced, on the external circle, the measured animal paths that result to be nearly independent of the entrance angle. Nevertheless, the effect of such input is nearly lost for $d>\ell _{MFP}$ when the input profile in the domain is nearly Lambertian, as tested by dedicated analysis of the animal paths (see Fig. 1 in the supplemental materials). The discrepancy of the $\langle L\rangle$ values with respect to the IP shown in Fig. 2 is then not due to a non-Lambertian input. This point will be clarified in the following. As a further characterization, in Fig. 3 is reported the density of trajectories of the studied system.

The link between validity of IP and constancy of the trajectories density emerges from the data. The trajectories density is variable inside the domain showing that our open system with natural animal paths does not present a constant value of this quantity, while the constancy of the characteristics quantities of the walkers movement is indeed required for the validity of the IP.

It is known that, in case of Lambertian input on the external boundary, if synthetic walkers have an exponential (also known as Lambert-Beer) path step distribution (see supplemental material for a detailed description), then the walker density inside the domain is constant for any geometry and inhomogeneities of the domain^40^ and then Eq. (1) is rigorously valid. Such a characteristic has been recently proposed as a powerful and robust method to check the accuracy of a Monte Carlo computation^43^.

It is interesting now to test $\langle L\rangle$ in a simulation where we replace the actual animal trajectories with synthetic walkers following an exponential path step distribution, with the same $\ell _{MFP}$ = 50 mm and maintaining the same isotropic input characteristics of the actual system (Fig. 1). The angles of the turning point is drawn from a uniform distribution between 0 and $2\pi$, since the angular distribution does not affect the validity of the IP^40,43^. The results of Fig. 4a show that the IP is substantially valid when $d=R_e-R > \ell _{MFP}$. In fact in such region $\langle L\rangle$ differs by less than 0.23% from $\langle L\rangle _{IP}$. These results can be assumed as an evaluation of the effect on $\langle L\rangle$ of a non-pure Lambertian entrance of the walkers, confirming that such an effect is very small. The results give us also an important indication for the understanding of the topic under investigation: the distribution of path steps of the animals play a critical role in the validity of Eq. (1). Even if simulated in an open system, an exponential step distribution ensures, with a good approximation, if $d > \ell _{MFP}$, the validity of the IP. Moreover, it is worth to stress that we performed simulations, reported in the supplemental material, with walkers following various path step distributions in our open system: the exponential is the only one that guarantees the fulfilment of the IP. The strict link between the validity of the IP and the constancy of the density of the walkers trajectories is confirmed in the result reported in Fig. 4, where the density of walker trajectories is in fact constant within 1.2% for $(R_e-r) > \ell _{MFP}$. The simulated results of Fig. 4a,b, compared to those of Figs. 2 and 3, suggest that the path step distribution of mollusks deviates from the exponential path step distribution.

Finally, it is interesting here to compare our results to those of Frangipane et al., where the movement patterns of the bacterium *Escherichia coli* satisfied the IP^46^. The actual reasons for such a difference are yet to be understood, and go beyond the aims of this paper. Beyond the obvious differences in the complexity of the studied organisms, one possible explanation is to be seen in the difference in the studied environment: the open system in our study is in contrast to the enclosed basin in their study, where a statistical equilibrium is likely to be present.

## Discussion

Using actual movements of animals registered in nature, we have analysed the mean path length $\langle L\rangle$ within a virtual medium in an open environment. Such an investigation has brought, for the first time to our knowledge, into focus the validity of the IP involving complex entities that perform random walks in an open environment.

In our study, based on chilton *Acanthopleure granulate* movements, the total mean path inside the medium $\langle L\rangle$ departs from the value $\langle L\rangle _{IP}$ predicted by the invariance property when the radius of the analysed dominium becomes larger than $\ell _{MFP}$, i.e., the mean free path between two consecutive decisive changes in direction. We ascribe the results to the condition of non equilibrium of the walkers that is reflected in the non constancy of the density of trajectory of the walker: when such quantity is not constant the IP condition is not respected. Moreover, the characteristics of the studied animals, characterized by a certain level of complexity and able to perform random walks during the foraging behavior, can play also a role in the non validity of the IP. In fact, a synthetic simulation of the walkers, made with an exponential path distribution, is, even in an open system, basically in agreement with the IP.

Our observation can give alternative information on the characteristics of animal behaviour in natural open systems with respect to step distribution and density. Finally, we observed deviation from the IP in an open environment, and although we cannot fully exclude that other species might display the IP, we expect it to be unlikely. However, further studies on other species and other habitats are needed to fully clarify whether this result is generalizable. Moreover, since it is conceptually relevant to demonstrate the eventual presence of IP in biological motion we present here a clear methodology, which may allow movement ecologists to test this hypothesis of invariance.

## Methods

Foraging excursions were recorded at Maycocks Bay (Barbados, West Indies) in August 1985. Chitons were equipped with a light-emitting-diode (LED) powered by a 1.5 V battery encased in dental acrylic at stuck on the second shell plate. Each chiton was also tagged with a numbered plastic label. Reference LEDs were positioned to facilitate path digitization. During nocturnal low tides the movements of animals were continuously recorded by a Robot Star 50 camera placed on the overhanging rocky cliff and triggered by a timer as described in Chelazzi et al.^49^. The method allowed to obtain the continuous recording of animal movements, without any error or uncertainty in the location of the animal at a given time. The continuous trajectories were then redrawn on paper and digitized, taking care of reporting all the visible changes of position. It is worth to note that the paths registered have been used for the results presented in this paper without any assumptions or arbitrariness in the conversion of animal path in steps. To determine the characteristic mean free path $\ell _{MFP}$, the acquired trajectories were discretized following the approach proposed by Humphries et al. et al.^48^. With this method the movement patterns are first projected onto the *x*- and *y*-axes to create two one-dimensional movement patterns for each individual. Turns in these projections can then be identified in an unambiguous way as occurring where the direction of travel changes.

During the observation campaign, a total of 113 trajectories have been recorded. In order to obtain a statistical significance of the calculated quantities a virtual environment is constructed as follows: the area of interest is a circle of radius *R*. The point coordinates of each trajectory are translated in order to obtain a common starting point, situated at a distance $R_e$ from the centre of the area of interest. This is equivalent, for our circular symmetry, to a random starting point along a circle of radius $R_e$. Each trajectory is then rotated around its starting point by a random angle, and this new path is utilized to calculate the length of the path within the circle of interest. When a trajectory exits from the circle and then re-enters, it is counted as a new trajectory. To calculate the trajectories density, the circular area is divided into annular concentric regions of equal area and the path travelled in each of these regions is registered for every animal trajectory. The procedure is repeated a number of times of the order of 10$^8$. About the evaluation of the uncertainty, we decided, for the sake of clarity, to report them only in Fig. 2, taking into account the actual number of the different trajectories. The same geometry has been used for the simulation with synthetic walkers. In the results reported in the main text, the number of simulated walkers have been at least 10$^6$. All methods were carried out in accordance with relevant guidelines and regulations.

## Supplementary Information

Supplementary Information.

## Data Availability

The datasets used and/or analysed during the current study can be made available by the corresponding author on reasonable request.
