# Supplementary material for: On the mean path length invariance property for random walks of animals in open environment
Source: Sci Rep. 2022 Nov 17;12:19800. doi: 10.1038/s41598-022-24361-9 (PMC9672306; doi:10.1038/s41598-022-24361-9)
Supplement: Supplementary file 1 — Supplementary Information. [file 41598_2022_24361_MOESM1_ESM.pdf]

On the mean path length invariance property for random walks of animals in open environment: Supplementary Material

Federico Tommasi[1,*], Lorenzo Fini[1], Stefano Focardi[2], Fabrizio Martelli[1], Giacomo Santini[3], & Stefano Cavalieri[1]*federico.tommasi@unifi.it

[1]*Dipartimento di Fisica e Astronomia, Università di Firenze, via Giovanni Sansone 1, I-50019, Sesto Fiorentino, Italy*

[2]*Istituto dei Sistemi Complessi, Consiglio Nazionale delle Ricerche, Via Madonna del piano 10, I-50019, Sesto Fiorentino, Italy*

[3]*Dipartimento di Biologia, Università di Firenze, Via Madonna del piano 6, I-50019, Sesto Fiorentino, Italy*

[*]*federico.tommasi@unifi.it*

Introduction

In this document we provide further information on the following points mentioned in the main body of the article: 1) The angular distribution of the paths of animals entering the considered circle domain; 2) The effect of the step length distribution on the validity of the IP; 3) The Lambert-Beer (exponential) step length distribution. For the point 1 we have plotted the entrance angular distribution of the measured animal paths. For the point 2 we have considered the average path length calculated for synthetic walkers by using three different kinds of step length distribution

such as exponential (Lambert-Beer), Pareto and Weierstrass. For the point 3 we have provided a synthetic description of the Lambert-Beer distribution.

Results

In Fig. 1 the entrance angular distribution of the measured animal paths through the considered circle domain is plotted for different values of the distance d. The results show that when the distance increases the angular distribution tends to become equal to the Lambertian angular distribution.

In Fig. 2 the average path length $\langle L \rangle$ spent by synthetic walkers inside the inner circle of radius R is plotted normalized to the IP value. Three different path length distributions with the same mean free path (50 mm) are used: exponential, Pareto (with a power law having power exponent equal to 1.1) and four modes-Weierstrass (with equivalent tail of the Pareto). The results in figure show that only for the exponential distribution we have a matching with the IP values for almost all values considered for R. Whilst for Pareto and Weierstrass we have the tendency to match the IP values only for small R values. The exponential distribution is usually known as Lambert-Beer distribution. The expression of the probability density function $p_{LB}(\ell)$ of this distribution is:

$$p_{LB}(\ell) = \frac{e^{-\frac{\ell}{\ell_{MFP}}}}{\ell_{MFP}}, \tag{1}$$

where ℓ_{MFP} is the mean free path for the random walkers. The origin of the Lambert-Beer distribution, i.e., Eq. (1), is from classical radiative transfer where the step length distribution of photons is expected to be exponential. The Lambert-Beer distribution implies a memoryless propagation

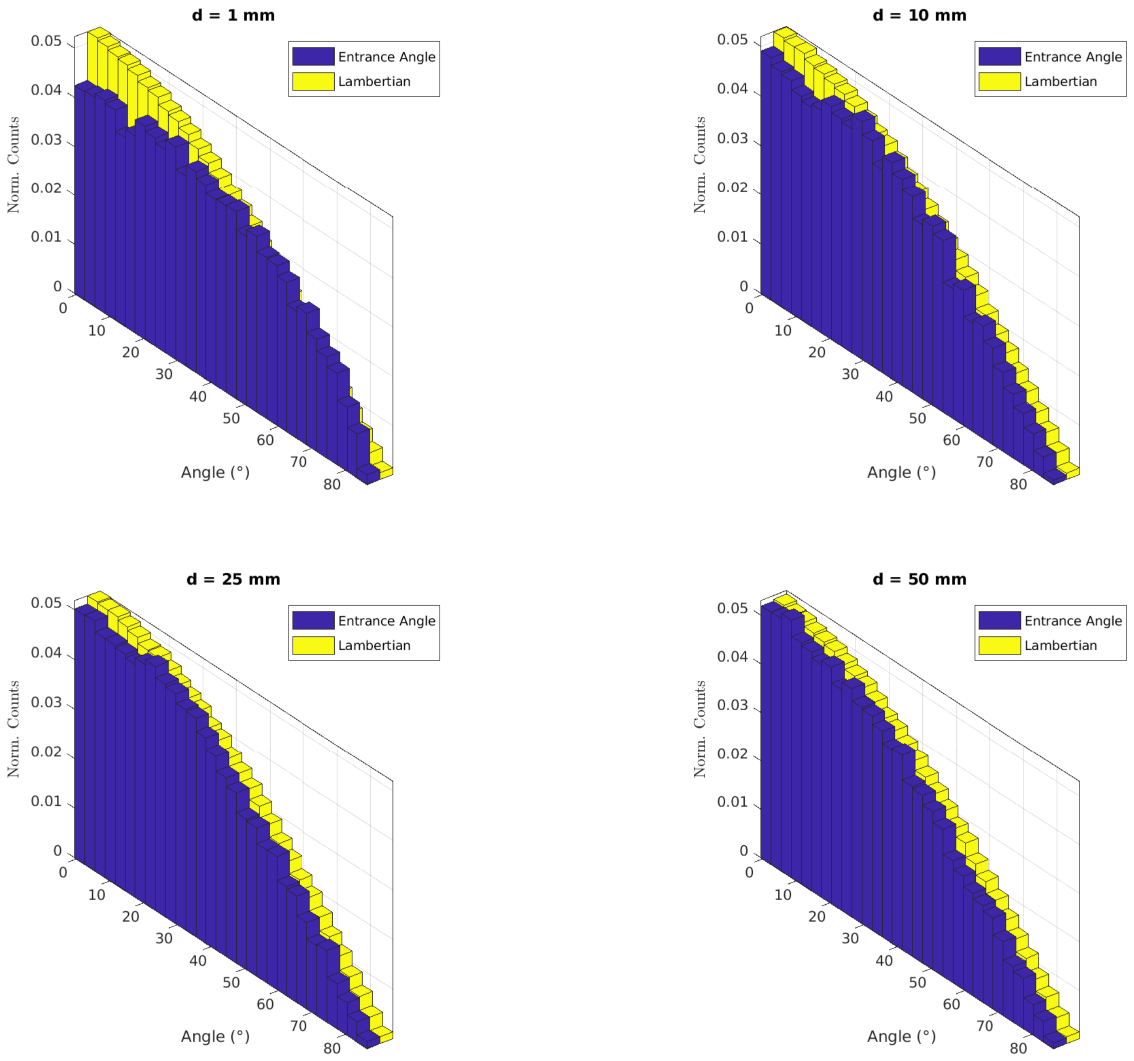

Figure 1: Angular distribution at the entrance of the circle for the measured animal paths shown for $d = 1,\ 10,\ 25,\ and\ 50\,mm$*. The Lambertian profile is also reported.*

so that the changes of directions are statistically uncorrelated. This distribution is, indeed, the most used in many fields of scientific research. For instance, Lambert-Beer is the standard distribution for transport phenomena in fields such as light transport in general, neutron transport, tissue optics.

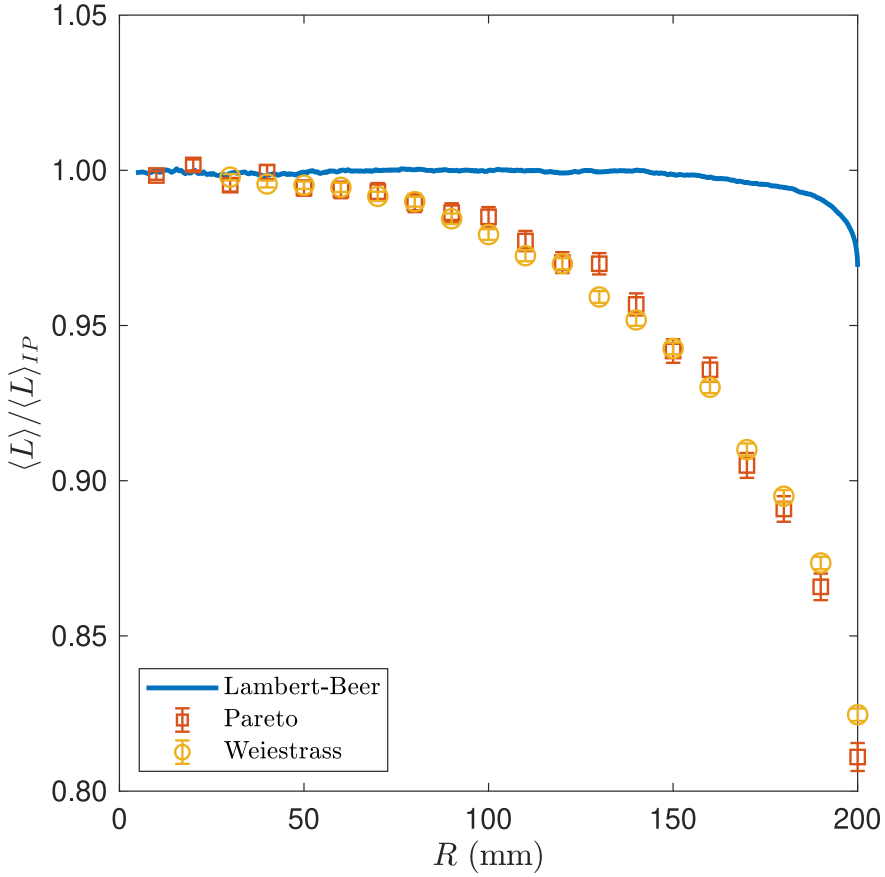

Figure 2: Average path length spent by synthetic walkers inside the inner circle of radius R normalized to the IP value. The value of the corresponding d is on the top x-axis. Three different distributions with the same mean free path (50 mm) are used: exponential (Lambert-Beer), Pareto ($\alpha = 1.1$) and four modes-Weierstrass.
